# Dimethyl 6-iodo-2-methyl-1,2-di­hydro­quinoline-2,4-di­carboxyl­ate

**DOI:** 10.1107/S1600536813023544

**Published:** 2013-08-31

**Authors:** Zeynep Gültekin, Wolfgang Frey, Tuncer Hökelek

**Affiliations:** a Department of Chemistry, Çankırı Karatekin University, TR-18100 Çankırı, Turkey; b Universität Stuttgart, Pfaffenwaldring 55, D-70569 Stuttgart, Germany; c Department of Physics, Hacettepe University, 06800 Beytepe, Ankara, Turkey

## Abstract

In the title compound, C_14_H_14_INO_4_, the di­hydro­pyridine ring adopts a twist conformation. In the crystal, pairs of N—H⋯O and C—H⋯O hydrogen bonds link the mol­ecules into inversion *R*_2_^2^(10) and *R*_2_^2^(18) dimers, forming infinite double chains running along the *c* axis.

## Related literature

For the conversion of 1,2-di­hydro­quinoline derivatives to the syntheses of quinolines, see: Dauphinee & Forrest (1978 ▶). For the conversion of 1,2-di­hydro­quinoline derivatives to the syntheses of 1,2,3,4-tetra­hydro­quinolines, see: Katritzky *et al.* (1996 ▶). For literature methods for the preparation of 1,2-di­hydro­quinolines, see: Dauphinee & Forrest (1978 ▶); Durgadas *et al.* (2010 ▶); Gültekin & Frey (2012 ▶); Makino *et al.* (2003 ▶); Yadav *et al.* (2007 ▶); Waldmann *et al.* (2008 ▶). For related structures, see: Gültekin *et al.* (2010 ▶, 2011*a* ▶,*b* ▶, 2012*a* ▶,*b* ▶). For ring puckering parameters, see: Cremer & Pople (1975 ▶). For hydrogen-bond motifs, see: Bernstein *et al.* (1995 ▶).
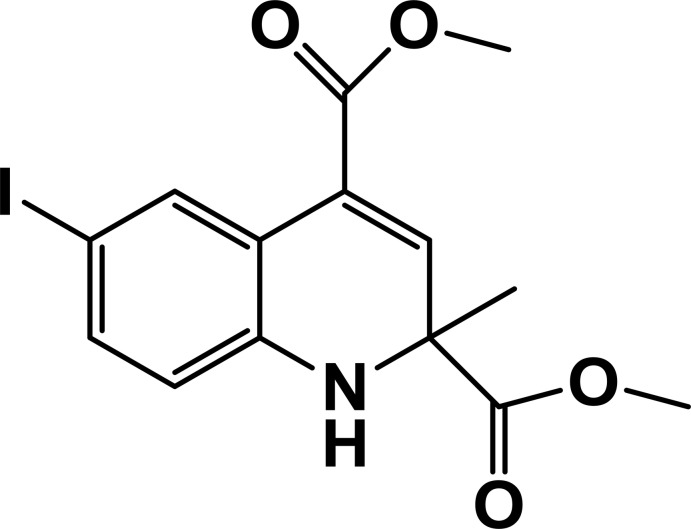


## Experimental

### 

#### Crystal data

C_14_H_14_INO_4_
*M* *_r_* = 387.16
Triclinic, 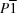

*a* = 7.7994 (14) Å
*b* = 10.2797 (8) Å
*c* = 10.8056 (8) Å
α = 116.862 (3)°
β = 103.956 (4)°
γ = 96.780 (4)°
*V* = 723.80 (16) Å^3^
*Z* = 2
Mo *K*α radiation
μ = 2.22 mm^−1^
*T* = 100 K
0.76 × 0.65 × 0.48 mm

#### Data collection

Bruker Kappa APEXII DUO diffractometer
Absorption correction: numerical (Blessing, 1995 ▶) *T*_min_ = 0.283, *T*_max_ = 0.415
43287 measured reflections
7044 independent reflections
6902 reflections with *I* > 2σ(*I*)
*R*_int_ = 0.032

#### Refinement

*R*[*F* ^2^ > 2σ(*F* ^2^)] = 0.027
*wR*(*F* ^2^) = 0.073
*S* = 1.24
7044 reflections
190 parameters
1 restraint
H atoms treated by a mixture of independent and constrained refinement
Δρ_max_ = 1.88 e Å^−3^
Δρ_min_ = −1.54 e Å^−3^

### 

Data collection: *APEX2* (Bruker, 2008 ▶); cell refinement: *SAINT* (Bruker, 2008 ▶); data reduction: *SAINT*; program(s) used to solve structure: *SHELXS97* (Sheldrick, 2008 ▶); program(s) used to refine structure: *SHELXL97* (Sheldrick, 2008 ▶); molecular graphics: *ORTEP-3 for Windows* (Farrugia, 2012 ▶); software used to prepare material for publication: *WinGX* (Farrugia, 2012 ▶) and *PLATON* (Spek, 2009 ▶).

## Supplementary Material

Crystal structure: contains datablock(s) I, global. DOI: 10.1107/S1600536813023544/gw2137sup1.cif

Structure factors: contains datablock(s) I. DOI: 10.1107/S1600536813023544/gw2137Isup2.hkl

Supplementary material file. DOI: 10.1107/S1600536813023544/gw2137Isup3.cml

Additional supplementary materials:  crystallographic information; 3D view; checkCIF report

## Figures and Tables

**Table 1:** Hydrogen-bond geometry (Å, °)

*D*—H⋯*A*	*D*—H	H⋯*A*	*D*⋯*A*	*D*—H⋯*A*
N1—H1⋯O3^i^	0.82 (2)	2.17 (2)	2.9795 (19)	169 (2)
C5—H5⋯O1	0.95	2.22	2.866 (2)	125
C12—H12*C*⋯O1^ii^	0.98	2.46	3.132 (2)	126

Symmetry codes: (i) 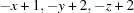; (ii) 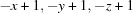.

**Fig. 1.**
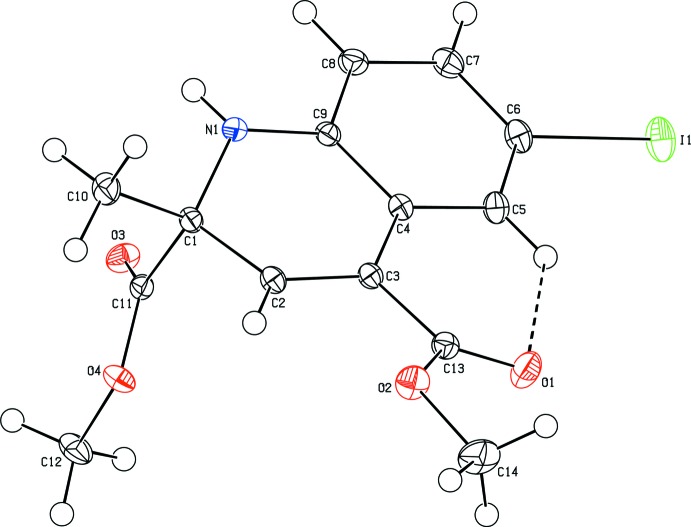
The molecular structure of the title compound with the atom-numbering scheme. Displacement ellipsoids are drawn at the 50% probability level. Hydrogen bond is shown as dashed line.

**Fig. 2.**
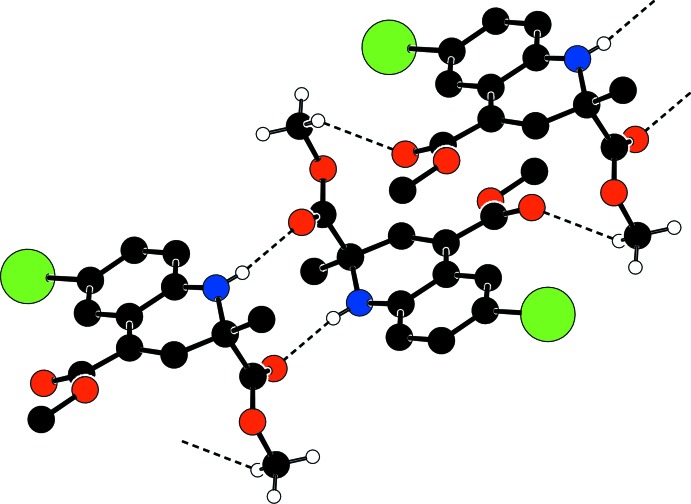
A partial packing diagram. Hydrogen bonds are shown as dashed lines. Hydrogen atoms not involved in hydrogen bonding have been omitted for clarity.

## supplementary crystallographic information

### 1. Comment

1,2-Dihydroquinoline derivatives are important for the preparation of pharmaceuticals and other biologically active compounds, and they are versatile intermediates in organic chemistry. They are converted to the syntheses of quinolines (Dauphinee & Forrest, 1978) and 1,2,3,4-tetrahydroquinolines (Katritzky *et al.*, 1996). Several methods have been described for the syntheses of 1,2-dihydroquinolines (Durgadas *et al.*, 2010; Makino *et al.*, 2003; Yadav *et al.*, 2007; Dauphinee & Forrest, 1978; Waldmann *et al.*, 2008).

The structures of some 1,2-dihydroquinoline derivatives, C_16_H_19_NO_4_ (Gültekin *et al.*, 2010), C_14_H_15_NO_4_ (Gültekin *et al.*, 2011*a*), C_17_H_21_NO_7_ (Gültekin *et al.*, 2011*b*), C_16_H_17_NO_5_ (Gültekin *et al.*, 2012*a*) and C_14_H_14_BrNO_4_ (Gültekin *et al.*, 2012*b*) have also been determined.

In the title compound (Fig. 1), the ring A (C1-C4/C9/C10/N1) is not planar, but adopting a twisted conformation with puckering parameters (Cremer & Pople, 1975) Q_T_ = 0.332 (1)Å, φ = 35.3 (3)° and θ = 66.0 (2)°. Ring A has a pseudo two-fold axis running through the midpoints of the N1—C1 and C3—C4 bonds.

In the crystal structure, intermolecular N—H···O and C—H···O hydrogen bonds (Table 1) link the molecules into centrosymmetric R_2_^2^(10) and R_2_^2^(18) dimers, respectively (Bernstein *et al.*, 1995) (Fig. 2), to form infinite double chains running along the c-axis.

### 2. Experimental

The title compound was synthesized by the literature method (Gültekin & Frey, 2012; Waldmann *et al.*, 2008). p-iodo aniline (100 mg, 1 eq) was dissolved in acetonitrile (1.5 ml), and then Bi(OTf)_3_ (5 mol%, 0.05 eq) and methyl pyruvate (2.2 eq) were added to the mixture. The mixture was heated by microwave irradiation for 7 h until the starting material was completely consumed as monitored by TLC. The resultant residue was directly purified by flash chromatography on silica (EtOAc:Cylohexane 1:2). Recrystallization over pentane and ethyl acetate (70:30) gave a yellow crystalline solid (yield: 34%), R_f_ 0.56 (2:1 Cyclohexane/EtOAc) m.p.: 393 K.

### Crystal data

**Table:**

C_14_H_14_INO_4_	*Z* = 2
*M**_r_* = 387.16	*F*(000) = 380
Triclinic, *P*1	*D*_x_ = 1.776 Mg m^−^^3^
Hall symbol: -P 1	Mo *K*α radiation, λ = 0.71073 Å
*a* = 7.7994 (14) Å	Cell parameters from 6892 reflections
*b* = 10.2797 (8) Å	θ = 2.3–36.5°
*c* = 10.8056 (8) Å	µ = 2.22 mm^−^^1^
α = 116.862 (3)°	*T* = 100 K
β = 103.956 (4)°	Block, yellow
γ = 96.780 (4)°	0.76 × 0.65 × 0.48 mm
*V* = 723.80 (16) Å^3^	

### Data collection

**Table:**

Bruker Kappa APEXII DUO diffractometer	7044 independent reflections
Radiation source: fine-focus sealed tube	6902 reflections with *I* > 2σ(*I*)
Triumph monochromator	*R*_int_ = 0.032
φ and ω scans	θ_max_ = 36.5°, θ_min_ = 2.3°
Absorption correction: numerical (Blessing, 1995)	*h* = −13→13
*T*_min_ = 0.283, *T*_max_ = 0.415	*k* = −17→17
43287 measured reflections	*l* = −18→18

### Refinement

**Table:**

Refinement on *F*^2^	Secondary atom site location: difference Fourier map
Least-squares matrix: full	Hydrogen site location: inferred from neighbouring sites
*R*[*F*^2^ > 2σ(*F*^2^)] = 0.027	H atoms treated by a mixture of independent and constrained refinement
*wR*(*F*^2^) = 0.073	*w* = 1/[σ^2^(*F*_o_^2^) + (0.0263*P*)^2^ + 0.7072*P*] where *P* = (*F*_o_^2^ + 2*F*_c_^2^)/3
*S* = 1.24	(Δ/σ)_max_ < 0.001
7044 reflections	Δρ_max_ = 1.88 e Å^−^^3^
190 parameters	Δρ_min_ = −1.54 e Å^−^^3^
1 restraint	Extinction correction: *SHELXL97* (Sheldrick, 2008), Fc^*^=kFc[1+0.001xFc^2^λ^3^/sin(2θ)]^-1/4^
Primary atom site location: structure-invariant direct methods	Extinction coefficient: 0.0715 (18)

### Special details

**Table:**

Geometry. All esds (except the esd in the dihedral angle between two l.s. planes) are estimated using the full covariance matrix. The cell esds are taken into account individually in the estimation of esds in distances, angles and torsion angles; correlations between esds in cell parameters are only used when they are defined by crystal symmetry. An approximate (isotropic) treatment of cell esds is used for estimating esds involving l.s. planes.
Refinement. Refinement of F^2^ against ALL reflections. The weighted R-factor wR and goodness of fit S are based on F^2^, conventional R-factors R are based on F, with F set to zero for negative F^2^. The threshold expression of F^2^ > 2sigma(F^2^) is used only for calculating R-factors(gt) etc. and is not relevant to the choice of reflections for refinement. R-factors based on F^2^ are statistically about twice as large as those based on F, and R- factors based on ALL data will be even larger.

### Fractional atomic coordinates and isotropic or equivalent isotropic displacement parameters (Å^2^)

**Table:**

	*x*	*y*	*z*	*U*_iso_*/*U*_eq_	
I1	0.017285 (15)	0.670510 (12)	0.215800 (11)	0.02370 (4)	
O1	0.28824 (19)	0.31504 (13)	0.37768 (13)	0.0217 (2)	
O2	0.34912 (17)	0.25624 (12)	0.55598 (13)	0.01879 (18)	
O3	0.69165 (15)	0.92071 (11)	0.93569 (13)	0.01678 (17)	
O4	0.78140 (13)	0.71616 (12)	0.92154 (12)	0.01509 (16)	
N1	0.32777 (15)	0.77436 (13)	0.85280 (12)	0.01270 (17)	
H1	0.335 (4)	0.858 (2)	0.920 (2)	0.020 (6)*	
C1	0.46679 (16)	0.70426 (14)	0.88944 (13)	0.01127 (17)	
C2	0.42693 (16)	0.54604 (14)	0.76450 (14)	0.01201 (18)	
H2	0.472 (3)	0.475 (3)	0.787 (3)	0.013 (5)*	
C3	0.33684 (16)	0.50661 (14)	0.62481 (14)	0.01130 (17)	
C4	0.26466 (16)	0.61703 (14)	0.59074 (14)	0.01143 (17)	
C5	0.19306 (18)	0.59520 (15)	0.44880 (15)	0.01460 (19)	
H5	0.1942	0.5060	0.3664	0.018*	
C6	0.12035 (19)	0.70379 (16)	0.42832 (15)	0.0157 (2)	
C7	0.11561 (19)	0.83472 (16)	0.54662 (16)	0.0165 (2)	
H7	0.0636	0.9073	0.5310	0.020*	
C8	0.18759 (18)	0.85846 (15)	0.68790 (15)	0.0149 (2)	
H8	0.1851	0.9481	0.7693	0.018*	
C9	0.26382 (16)	0.75191 (14)	0.71195 (14)	0.01145 (17)	
C10	0.47035 (19)	0.70673 (18)	1.03339 (15)	0.0169 (2)	
H10A	0.4966	0.8115	1.1121	0.025*	
H10B	0.5656	0.6607	1.0599	0.025*	
H10C	0.3511	0.6496	1.0199	0.025*	
C11	0.65818 (16)	0.79393 (14)	0.91643 (13)	0.01153 (17)	
C12	0.96471 (18)	0.78904 (19)	0.94143 (18)	0.0191 (2)	
H12A	1.0454	0.7234	0.9438	0.029*	
H12B	1.0110	0.8854	1.0343	0.029*	
H12C	0.9615	0.8076	0.8597	0.029*	
C13	0.31983 (17)	0.35216 (14)	0.50561 (15)	0.01327 (19)	
C14	0.3488 (3)	0.10914 (18)	0.4461 (2)	0.0252 (3)	
H14A	0.3710	0.0458	0.4910	0.038*	
H14B	0.4454	0.1193	0.4050	0.038*	
H14C	0.2297	0.0621	0.3674	0.038*	

### Atomic displacement parameters (Å^2^)

**Table:**

	*U*^11^	*U*^22^	*U*^33^	*U*^12^	*U*^13^	*U*^23^
I1	0.02815 (6)	0.02630 (6)	0.01695 (5)	0.00288 (4)	0.00133 (4)	0.01504 (4)
O1	0.0310 (6)	0.0176 (4)	0.0144 (4)	0.0086 (4)	0.0071 (4)	0.0059 (4)
O2	0.0239 (5)	0.0128 (4)	0.0208 (5)	0.0062 (3)	0.0069 (4)	0.0093 (4)
O3	0.0174 (4)	0.0108 (4)	0.0201 (4)	0.0024 (3)	0.0085 (3)	0.0053 (3)
O4	0.0096 (3)	0.0185 (4)	0.0204 (4)	0.0040 (3)	0.0045 (3)	0.0124 (4)
N1	0.0118 (4)	0.0151 (4)	0.0112 (4)	0.0057 (3)	0.0040 (3)	0.0060 (3)
C1	0.0095 (4)	0.0137 (4)	0.0106 (4)	0.0023 (3)	0.0028 (3)	0.0065 (4)
C2	0.0111 (4)	0.0128 (4)	0.0128 (4)	0.0024 (3)	0.0030 (3)	0.0076 (4)
C3	0.0104 (4)	0.0116 (4)	0.0119 (4)	0.0021 (3)	0.0031 (3)	0.0063 (4)
C4	0.0106 (4)	0.0121 (4)	0.0117 (4)	0.0024 (3)	0.0028 (3)	0.0066 (4)
C5	0.0158 (5)	0.0150 (5)	0.0125 (5)	0.0025 (4)	0.0028 (4)	0.0078 (4)
C6	0.0162 (5)	0.0177 (5)	0.0149 (5)	0.0033 (4)	0.0025 (4)	0.0109 (4)
C7	0.0163 (5)	0.0184 (5)	0.0186 (5)	0.0068 (4)	0.0048 (4)	0.0123 (5)
C8	0.0149 (5)	0.0159 (5)	0.0163 (5)	0.0071 (4)	0.0056 (4)	0.0090 (4)
C9	0.0097 (4)	0.0134 (4)	0.0119 (4)	0.0033 (3)	0.0036 (3)	0.0068 (4)
C10	0.0163 (5)	0.0237 (6)	0.0134 (5)	0.0042 (4)	0.0055 (4)	0.0114 (5)
C11	0.0108 (4)	0.0129 (4)	0.0098 (4)	0.0025 (3)	0.0035 (3)	0.0049 (4)
C12	0.0099 (4)	0.0269 (6)	0.0230 (6)	0.0032 (4)	0.0048 (4)	0.0149 (5)
C13	0.0123 (4)	0.0122 (4)	0.0143 (5)	0.0025 (3)	0.0039 (4)	0.0062 (4)
C14	0.0297 (7)	0.0140 (5)	0.0318 (8)	0.0092 (5)	0.0120 (6)	0.0095 (5)

### Geometric parameters (Å, º)

**Table:**

I1—C6	2.0908 (14)	C4—C9	1.4154 (17)
N1—C1	1.4447 (17)	C5—C6	1.3888 (19)
N1—C9	1.3812 (17)	C5—H5	0.9500
N1—H1	0.821 (17)	C6—C7	1.388 (2)
O1—C13	1.2059 (17)	C7—C8	1.387 (2)
O2—C13	1.3412 (17)	C7—H7	0.9500
O2—C14	1.439 (2)	C8—C9	1.3989 (18)
O3—C11	1.2075 (16)	C8—H8	0.9500
O4—C11	1.3273 (16)	C10—H10A	0.9800
O4—C12	1.4508 (17)	C10—H10B	0.9800
C1—C2	1.5028 (18)	C10—H10C	0.9800
C1—C10	1.5376 (18)	C12—H12A	0.9800
C1—C11	1.5444 (17)	C12—H12B	0.9800
C2—C3	1.3435 (18)	C12—H12C	0.9800
C2—H2	0.94 (2)	C14—H14A	0.9800
C3—C4	1.4728 (17)	C14—H14B	0.9800
C3—C13	1.4890 (18)	C14—H14C	0.9800
C4—C5	1.4002 (18)		
			
C13—O2—C14	114.26 (13)	C7—C8—H8	119.6
C11—O4—C12	115.43 (11)	C9—C8—H8	119.6
C1—N1—H1	115 (2)	N1—C9—C4	120.20 (11)
C9—N1—C1	120.03 (10)	N1—C9—C8	119.93 (11)
C9—N1—H1	116 (2)	C8—C9—C4	119.75 (11)
N1—C1—C2	109.26 (10)	C1—C10—H10A	109.5
N1—C1—C10	108.89 (10)	C1—C10—H10B	109.5
N1—C1—C11	110.73 (10)	C1—C10—H10C	109.5
C2—C1—C10	111.83 (11)	H10A—C10—H10B	109.5
C2—C1—C11	108.91 (10)	H10A—C10—H10C	109.5
C10—C1—C11	107.20 (10)	H10B—C10—H10C	109.5
C1—C2—H2	117.7 (15)	O3—C11—O4	124.68 (12)
C3—C2—C1	121.91 (11)	O3—C11—C1	123.96 (11)
C3—C2—H2	120.4 (15)	O4—C11—C1	111.31 (11)
C2—C3—C4	120.28 (11)	O4—C12—H12A	109.5
C2—C3—C13	118.60 (11)	O4—C12—H12B	109.5
C4—C3—C13	121.05 (11)	O4—C12—H12C	109.5
C5—C4—C3	124.75 (11)	H12A—C12—H12B	109.5
C5—C4—C9	118.89 (11)	H12A—C12—H12C	109.5
C9—C4—C3	116.33 (11)	H12B—C12—H12C	109.5
C4—C5—H5	119.9	O1—C13—O2	122.31 (13)
C6—C5—C4	120.11 (12)	O1—C13—C3	125.09 (12)
C6—C5—H5	119.9	O2—C13—C3	112.57 (11)
C5—C6—I1	119.55 (10)	O2—C14—H14A	109.5
C7—C6—I1	119.26 (10)	O2—C14—H14B	109.5
C7—C6—C5	121.19 (12)	O2—C14—H14C	109.5
C6—C7—H7	120.3	H14A—C14—H14B	109.5
C8—C7—C6	119.31 (12)	H14A—C14—H14C	109.5
C8—C7—H7	120.3	H14B—C14—H14C	109.5
C7—C8—C9	120.73 (12)		
			
C14—O2—C13—O1	3.0 (2)	C2—C3—C4—C9	−11.93 (17)
C14—O2—C13—C3	−175.15 (12)	C13—C3—C4—C5	−6.82 (18)
C12—O4—C11—O3	−4.60 (19)	C13—C3—C4—C9	171.05 (11)
C12—O4—C11—C1	177.95 (11)	C2—C3—C13—O1	−159.19 (14)
C9—N1—C1—C2	−40.61 (15)	C2—C3—C13—O2	18.92 (16)
C9—N1—C1—C10	−163.02 (11)	C4—C3—C13—O1	17.9 (2)
C9—N1—C1—C11	79.35 (14)	C4—C3—C13—O2	−164.00 (11)
C1—N1—C9—C4	28.53 (17)	C3—C4—C5—C6	176.90 (12)
C1—N1—C9—C8	−155.41 (12)	C9—C4—C5—C6	−0.92 (19)
N1—C1—C2—C3	28.17 (16)	C3—C4—C9—N1	−0.21 (17)
C10—C1—C2—C3	148.81 (12)	C3—C4—C9—C8	−176.28 (11)
C11—C1—C2—C3	−92.90 (14)	C5—C4—C9—N1	177.79 (11)
N1—C1—C11—O3	14.82 (17)	C5—C4—C9—C8	1.72 (18)
N1—C1—C11—O4	−167.70 (10)	C4—C5—C6—I1	179.18 (9)
C2—C1—C11—O3	134.99 (13)	C4—C5—C6—C7	−0.5 (2)
C2—C1—C11—O4	−47.53 (14)	I1—C6—C7—C8	−178.59 (10)
C10—C1—C11—O3	−103.84 (15)	C5—C6—C7—C8	1.1 (2)
C10—C1—C11—O4	73.64 (13)	C6—C7—C8—C9	−0.2 (2)
C1—C2—C3—C4	−3.42 (18)	C7—C8—C9—N1	−177.23 (12)
C1—C2—C3—C13	173.68 (11)	C7—C8—C9—C4	−1.15 (19)
C2—C3—C4—C5	170.20 (12)		

### Hydrogen-bond geometry (Å, º)

**Table:**

*D*—H···*A*	*D*—H	H···*A*	*D*···*A*	*D*—H···*A*
N1—H1···O3^i^	0.82 (2)	2.17 (2)	2.9795 (19)	169 (2)
C5—H5···O1	0.95	2.22	2.866 (2)	125
C12—H12*C*···O1^ii^	0.98	2.46	3.132 (2)	126

Symmetry codes: (i) −*x*+1, −*y*+2, −*z*+2; (ii) −*x*+1, −*y*+1, −*z*+1.
